# 3D micro-organisation printing of mammalian cells to generate biological tissues

**DOI:** 10.1038/s41598-020-74191-w

**Published:** 2020-11-10

**Authors:** Gavin D. M. Jeffries, Shijun Xu, Tatsiana Lobovkina, Vladimir Kirejev, Florian Tusseau, Christoffer Gyllensten, Avadhesh Kumar Singh, Paul Karila, Lydia Moll, Owe Orwar

**Affiliations:** 1 Fluicell AB, Flöjelbergsgatan 8C, 431 37 Mölndal, Sweden; 2 Department of Physiology and Pharmacology, Karolinska Intitutet, Solnavägen 1, 171 77 Solna, Sweden; 3 Cellectricon AB, Neongatan 4B, 431 53 Mölndal, Sweden

**Keywords:** Biological techniques, Biotechnology

## Abstract

Significant strides have been made in the development of in vitro systems for disease modelling. However, the requirement of microenvironment control has placed limitations on the generation of relevant models. Herein, we present a biological tissue printing approach that employs open-volume microfluidics to position individual cells in complex 2D and 3D patterns, as well as in single cell arrays. The variety of bioprinted cell types employed, including skin epithelial (HaCaT), skin cancer (A431), liver cancer (Hep G2), and fibroblast (3T3-J2) cells, all of which exhibited excellent viability and survivability, allowing printed structures to rapidly develop into confluent tissues. To demonstrate a simple 2D oncology model, A431 and HaCaT cells were printed and grown into tissues. Furthermore, a basic skin model was established to probe drug response. 3D tissue formation was demonstrated by co-printing Hep G2 and 3T3-J2 cells onto an established fibroblast layer, the functionality of which was probed by measuring albumin production, and was found to be higher in comparison to both 2D and monoculture approaches. Bioprinting of primary cells was tested using acutely isolated primary rat dorsal root ganglia neurons, which survived and established processes. The presented technique offers a novel open-volume microfluidics approach to bioprint cells for the generation of biological tissues.

## Introduction

Our understanding of disease, as well as the action of new drugs, would dramatically increase, if it was easier to obtain early access to patient-relevant tissues, presenting the full disease phenotype rather than a cell line expressing key features. Disease etiology typically involves several different cell types, each playing a specific role in the development and rate of disease progression, as well as prognosis and therapeutic outcome. Recent studies have shown that different cell types found in tumor microenvironments are important in modulating tumor biology, as well as in response to drug compounds. Fibroblasts and endothelial cells, are key elements in the tumor microenvironment, playing essential roles in signalling through the secretion of molecules which can influence cancer cell behavior^1^. Reconstructing the tumor microenvironment would offer novel insights into the mechanisms of cancer and address the relationship between tissue structure and function.

Furthermore, drug development across many therapeutic areas such as oncology and neurodegeneration would be accelerated by access to high-fidelity, translational in vitro models of disease^2^. We show herein that such model tissues can be, in principle, made from several relevant cell types in a time- and cost-effective manner. Regenerative medicine and transplantation therapy are other areas that would benefit from “on-demand” access to printed human tissues^3^. Even though fully functional engineered organs are yet to be realised, there remains significant potential in generating therapeutic tissues, including microtissues comprising of stem cells, for a variety of applications with less stringent functional specifications^4–6^. In addition to pharmaceutical and therapeutic usage, artificial human tissues would positively impact the cosmetics industry, where a ban on the usage of experimental animals is a major driver.

Taking into consideration the rationale outlined above, it is not surprising that there is a growing number of experimental approaches to building complex tissue-like structures. These approaches can be generalised into three families; spontaneous self-assembly methods, cell patterning approaches, and bioprinting strategies.

Spontaneous self-assembly methods such as organoids, multicellular cultures, as well as 3D culturing and co-culturing techniques, rely upon cell positioning to be guided by chemotactic means. However, in this approach cell arrangement is largely stochastic in nature and little control is conveyed over the final configuration and orientation of the cellular construct. Cells are seeded to a region and allowed to propagate, controlled soley by the environmental conditions imposed upon them.

Cell patterning technologies, which include micro-stamp transfer and surface functionalisation, rely on modifying a surface with a predetermined pattern, upon which cells are deposited and cultured. As with spontaneous self-assembly, cell positioning remains largely stochastic, however preferential adhesion occurs where the predetermined patterns are constructed.

Bioprinting strategies are emerging as a promising means of generating biological tissues, built upon a range of technologies including: extrusion-based^7–9^, inkjet^8,10,11^, laser-based^12^, and microfluidics-based^13^. Each approach has its own key benefit, with the central aim of directly patterning cells into a 2D or 3D arrangement, from which the cells can grow and establish interconnectivity. However, a general need to house the cells in a supporting medium, such as a gel, still remains, placing a restriction on the ability to control the location of each cell in the printed construct. This both limits early cell-to-cell interactions, and the control of their local environment^8,14,15^.

Here, we have developed a new microfluidic bioprinting technology (Biopixlar, Fluicell AB, Sweden), capable of precisely controlling the ratio and type of deposited cells, in addition to controlling their relative position to each other, without the restrictions of a supporting gel or medium. In principle, it is possible to generate arbitrary cell structures in 2D and 3D where the coordinates and phenotype are predetermined, and where these printed constructs have the capability to grow into confluent tissues.

## Results

**Figure 1:**
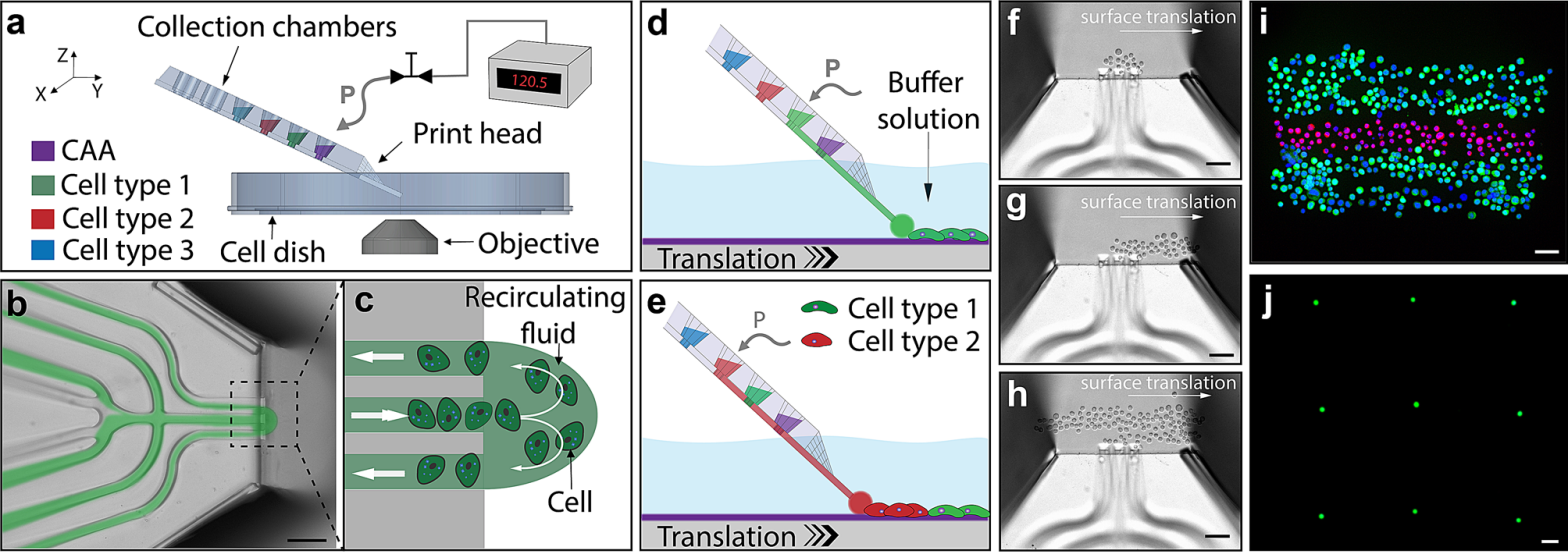
Introduction to the direct cell bioprinting approach, highlighting the key components. (**a**–**c**) illustrates the core concept of printing cells from a recirculating fluid flow. (**a**) Schematic overview of the bioprinting setup consisting of; a micro-positioner controlled printhead, an automated substrate positioner, a computer controlled pneumatic interface, and a microscopy imaging system. CAA refers to cell adhesion agent. (**b**) An overlay image combining fluorescence and brightfield modalities, demonstrating the fluidic printhead and the hydrodynamically confined flow. (**c**) An illustration of the fluidic printhead tip, presenting the flow path of cells within the confined circulating fluid. (**d**,**e**) illustrate the method of pattern formation using multiple cell types. (**d**) Positioning the printhead in proximity to a substrate surface within a buffer solution, whilst maintaining a confined fluid flow, enables cells to interact and attach to the substrate within a localised region at the tip of the printhead. Translating the surface relative to the printhead allows the deposited cells to be patterned. (**e**) Changing which solution within the printhead is actively being pressurised, allows numerous loaded cell types to be printed, without the need to replace or reload the printhead. (**f**–**h**) Experimental brightfield images demonstrate the formation of a structured pattern composed of alternating stripes of HaCaT and A431 cells onto a petri dish surface immersed in DMEM growth media. (**f**) presents HaCaT cells within the confined flow at the beginning of a print. (**g**) shows the first stripe of the pattern being printed. (**h**) presents the image mid-way through second pass of the HaCaT cell stripe. (**i**) presents a three-line structure composed of alternating printed stripes of HaCaT and A431 cells, labelled with cytotracker-green and cytotracker-red respectively. This structure was post-print nuclei stained with Hoechst 33342 and fixed with paraformaldehyde. A demonstration of the printing precision is shown in (**j**), whereby a single cell array was constructed using A431 cells. The scale bars represent 100 µm.

Our bioprinting approach utilises a recirculating fluid flow, generated at the tip of a free-standing microfluidic device^16,17^, which we use as a printhead to achieve high-resolution printing. This device was previously optimized for generating a hydrodynamically confined flow, which enabled contamination-free delivery of one miscible liquid inside another. As illustrated in Fig. 1a, the printhead has three independent cell chambers (in addition to one chamber for cell attachment agent (CAA), or washing solution) and is mounted to a high-resolution micro-positioner. The tip of the printhead can be positioned in three dimensions (e.g., with respect to both the lateral and axial location of the cells, indicated by the XYZ icon in Fig. 1a). A photograph of a loaded printhead (top and side view), where cell chambers were loaded with colored solutions for visualization purpose can be seen in the Supporting Information. Translating the surface relative to the printhead (typical translation rate is 30–50 µm/s), while controlling the recirculating fluid flow, allows cell patterns to be written with arbitrary geometries. A photomicrograph of the printhead tip is shown in Fig. 1b, where recirculating fluid flow carrying cells to be presented to the surface are shown schematically in Fig. 1c. Cells are confined to the recirculatory flow until they interact strongly enough with the surface to attach. This enables precise control over the cell patterning, even though both the cells and the printing substrate are immersed in miscible media. The printing surface was optimised for efficient cell adhesion by applying CAA solution, which can be deposited prior to the printing operation in a bulk format, or patterned directly prior to cell deposition (as described in the methods section). Employing a recirculatory mode of printing enables collection of any unattached cells, due to the orientation of the microfluidic channels; the delivery channel is, on either side, adjacent to a channel under negative pressure (Fig. 1c), such that any unattached cells are aspirated back into the printhead and transported into the onboard collection chambers.

To generate viable printed cell structures and to minimise cell sedimentation and aggregation within the chambers of the printhead, we housed the cells to be deposited in a cell culture media, supplemented with a polyethylene glycol solution (PEG, mol. wt. 6000), to a final concentration of 15 mg/ml. The substrate surface was submerged into the same culture media, without PEG. The printhead was maintained at a vertical displacement from the surface of approximately 10 µm, and the microscope stage was translated in the desired direction while maintaining circulation of cells at the tip of the printhead (Fig. 1d, Video S1). During the print run, the choice of cell type can be adjusted by controlling the pressures within the printhead. The selected chamber, housing the desired cell type, can be pressurised to drive the cell suspension to the surface. Through careful pressure balancing, the choice of cell to be deposited can be controlled, allowing real-time choice of cell pattern densities and cell ratio (Fig. 1e). Brightfield microscopy images of an example structure being printed are shown in Fig. 1f–h, demonstrating the evolution over time of alternating stripes of HaCaT (cytotracker-green stained) and A431 (cytotracker-red stained) cells onto a petridish surface immersed in DMEM culture media. The HaCaT cell stripe was printed first, where Fig. 1h presents an image mid-way through the printing process of a second pass, illustrating the accuracy and build up of printed regions using a multiple pass approach. This structure was post-print nuclei stained with Hoechst 33342 and fixed with paraformaldehyde. A fluorescence microscopy image of this final printed structure, composed of alternating printed stripes of HaCaT-A431-HaCaT cells, is shown in Fig. 1i.

The printhead can be loaded with 25 µl of both, high and low cell density solutions, allowing cells to be printed very close to each other, as well as to pattern individual cells into defined arrays, respectively. Individual cells can be reliably deposited, or groups of cells (Figs. 1j, S1), with a lateral resolution on the order of a cell width (e.g. less than 30 µm). A demonstration of this printing precision is shown in Fig. 1j, whereby a single cell array was constructed using A431 cells with a constant grid size of approximately 500 µm. To generate such a single cell array, a low cell density solution (ca. 2 × 10^6^ cells/ml versus typical ca. 8 × 10^6^ cells/ml) was used in combination with a pulsed mode of the bioprinting protocol, where a pulsed pressure was applied to the selected chamber (instead of continuous pressure supply). This allowed for the ejection of a single cell from the printhead tip, and after successful cell attachment to a surface, the substrate was translated to a new printing location. Printing precision was further investigated (see Supplementary Information, Fig. S1) by establishing a repeatable deposition of cell spot arrays. Through automated control of the substrate positioning, the spacing between cell spots can be fixed or adjusted in real time, enabling complex cell patterning whilst monitoring cell attachment.

We next examined the possibility to develop high-resolution cell prints into 2D confluent tissues, where cells proliferate and establish contact after incubation. To make defect-free tissues, long-term survival of printed cells is required. To investigate this aspect, we measured first the survivability of A431 and HaCaT cells after printing to ensure that shear forces and handling of cells through the microfluidic channels were not detrimental to cell viability. We found that the initial cell viabilities, 2 h post-printing, were 96% and 97% respectively. 24-h survivability studies of A431 and HaCaT cells both showed a survival rate in excess of 99%, indicating very minimal interference of the micropatterning approach on the overall health of the cells. Additional cell lines were probed for their viability after printing, example images and a summary of the results can be found in the Supplementary Information S3 and Table S1.

**Figure 2:**
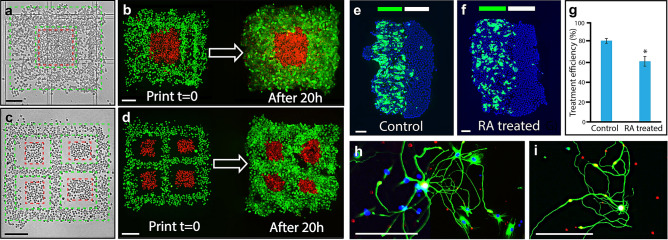
Post-printing model development and cell growth. (**a**–**d**) present experimental examples of an oncology model, highlighting before and after a 20 h incubation. A simple encapsulated islet of skin cancer cells (A431) surrounded by, and in contact with, an epithelial cell layer (HaCaT) is shown in (**a**,**b**) whereby HaCaT and A431 cells were labelled with cytotracker-green (green dashed boundary) and cytotracker-red (red dashed boundary), respectively prior to printing. A brightfield image of the pattern directly after printing is shown in (**a**), where dashed lines indicate the boundaries of the printed cell types. The corresponding fluorescent image is shown on the left of panel (**b**), whereby the grown oncology model is presented to the right of panel (**b**). A more complex version of this encapsulation model is presented in (**c**,**d**) whereby four skin cancer cell islets (A431), are printed and surrounded by an epithelial cell layer (HaCaT). The two cell types were printed to not be in contact, to allow each cell type to attach to the surface and grow prior to interacting with each other. (**c**,**d**) were performed using the same timing and conditions as for (**a**,**b**). (**e**,**f**) present examples of the basic skin model, composed of a two-printed stripe structure of differentiated and non-differentiated HaCaT cells expressing different levels of CK10. (**e**) presents a control tissue and (**f**) presents tissue treated with all trans retinoic acid (RA). The green and white bands at the top of the panels indicate the approximate printed stripe width of differentiated and non-differentiated cells, respectively. A summary plot of the RA treatment is shown in (**g**), presenting the mean values of CK10 expression for both the controls (81.8%, n = 10) and the treated samples (61.4%, n = 16), demonstrating a statistically significant reduction of approximately 25% in CK10 expression. Error bars represent standard error. (**h**,**i**) Show acutely isolated primary rat dorsal root ganglia (DRG) neurons, which were patterned and allowed to form processes. Post printing, the sample was allowed to mature and grow for 9 days in standard neuronal culture conditions, where network establishment could be monitored. The scale bar in all panels represents 200 µm.

Simple oncology models having multiple cell types, were created by printing an encapsulated islet (or patch) of A431 skin cancer cells framed by, and in contact with, a layer of HaCaT skin epithelial cells, as illustrated in Fig. 2. HaCaT and A431 cells were labelled with cytotracker-green (green-dashed boundary) and cytotracker-red (red-dashed boundary) respectively prior to printing. The immediate printed structure with rounded cell morphology can be seen in Fig. 2a as a brightfield microscopy image, and in the left half of Fig. 2b in multicolour fluorescence. A more advanced model of this structure was established by printing four square patches of A431 cells (ca. 10 × 10 cells) framed by a ca. 10 cells wide layer of HaCaT cells. The framing of HaCaT cells was printed a few cell widths away from the A431 islets, to ensure colony growth of the same cell type before interacting with a second cell type. The printed structure having cells with rounded morphology can be seen in Fig. 2c in brightfield mode and in the left half of Fig. 2d in fluorescence mode. Both printed structures grew and proliferated to establish continuous tissues after a 20 h incubation (Fig. 2b,d). The A431 skin cancer cell islet morphology was generally well preserved, having a low level of defects in the developed tissues. It was found that cells, printed in close contact with neighbouring cells, have low initial propagation, which allows cell patterns to be programmed into the final tissue. Following this approach, we further investigated a printed skin model for functional testing through its response to all-trans retinoic acid (RA). RA is a natural regulator of epidermal proliferation and differentiation, and is commonly used for the treatment of various dermatological disorders^18,19^. To build this model, HaCaT cells were differentiated to represent the spinous epidermal layer of native skin, while non-differentiated HaCaT cells were used to represent the basal layer. These two cell types were co-printed in stripes side-by-side and allowed to grow to form contiguous tissues. The cells were found to be viable 24 h after printing, demonstrating proliferation while maintaining their differentiated state. Cytokeratin 10 (CK10) is expressed in the differentiated (spinous layer) HaCaT cells, distinguishing it with non-differentiated (basal layer) HaCaT cells^20^. Four hours post-printing, samples were exposed to RA-supplemented full growth media (to a final concentration 1 µM), whereas control samples were treated with an equal concentration of RA solvent (DMSO) in the same growth media for the following 20 h.

The effect of RA treatment on printed stripes was compared against a printed and grown control sample of similar dimensions. Fixed and stained examples of the models are presented in Fig. 2e,f, where green and white bands at the top of the printed regions are indicative of the approximate printed stripe width of differentiated and non-differentiated cells, respectively. A summary plot of the RA treatment is shown in (Fig. 2g), presenting the mean values of CK10 expression for both the controls (81.8%, n = 10) and the treated samples (61.4%, n = 16), demonstrating a statistically significant reduction in CK10 expression of approximately 25% (Student’s t-test, p-value 0.0003, 95% CI). To quantify CK10 expression, the total number of cells in differentiated region was estimated by counting DAPI stained nuclei in the blue channel, and the number of CK10-expressing cells were derived by overlaying and masking the blue and green areas. The threshold for the expression of CK10 was taken as the background noise level in the green fluorescence channel.

Although more physiologically relevant tissue models using cell lines should play an expanded role in future biological research, a critical need emanating from the medical and pharmaceutical fields is to generate tissues and disease models from small samples and patient biopsies. To examine biopsies as a cell source, we employed the microfluidic printhead, to pattern cells isolated from a primary neuronal tissue sample. Acutely isolated primary rat dorsal root ganglia neurons (DRGs) were microsurgically dissected from 5–7 weeks old male Sprague–Dawley rats as described elsewhere^21^. The printing substrate and the neuronal cell suspension were prepared similarly to the oncology model printing (details outlined in the methods section). An area of 1 × 1 mm was patterned and subsequently cultured for 9 days, before fixing and immunostaining. Selected regions of this sample are presented in Fig. 2h,i, demonstrating the ability of the printed cells to not only survive, but also to form processes. This initial demonstration of bioprinting primary neurons from a small acutely isolated sample, indicates the potential within disease model generation for studies of e.g. pain, neurodegenerative disease, and other CNS disorders.

**Figure 3:**
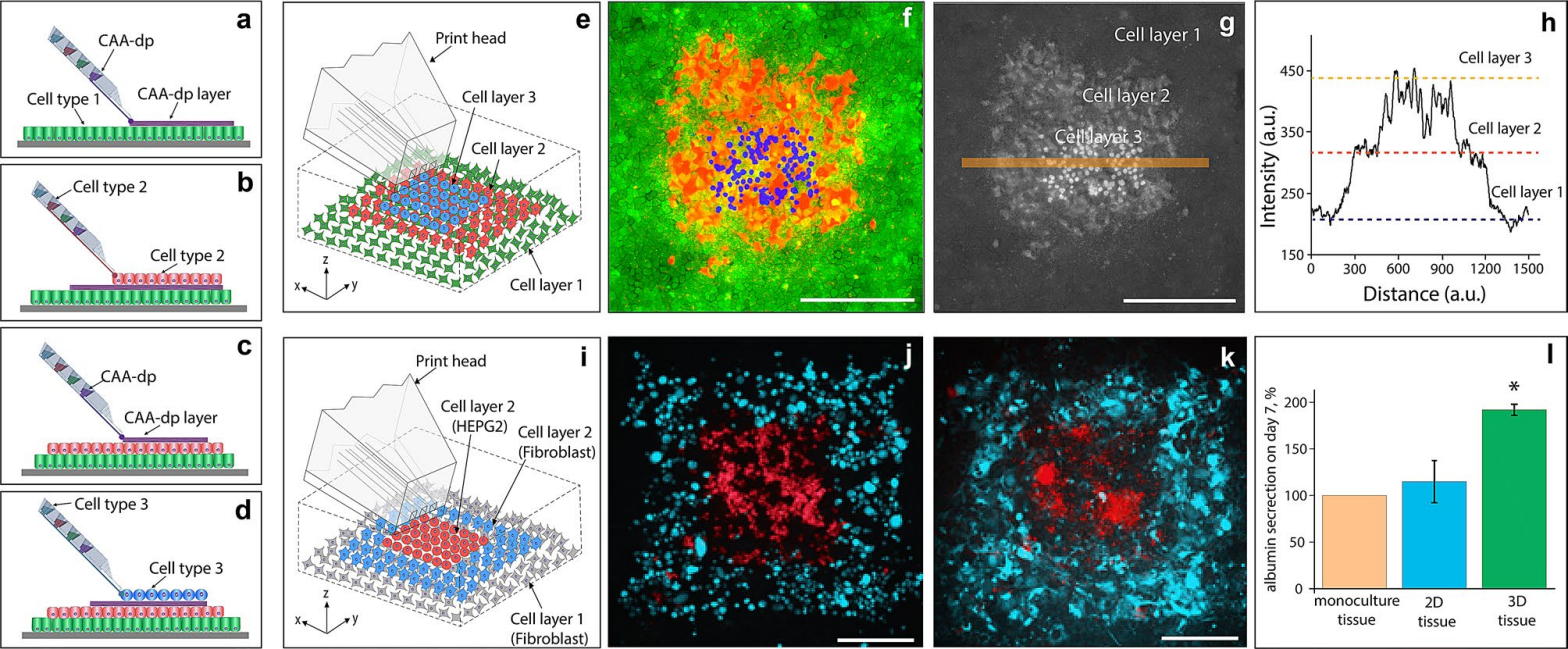
Direct 3D cell bioprinting (**a**–**d**) is an overview of the 3D printing process, whereby a substrate is pre-cultured with a confluent cell layer, upon which a layer of cell attachment agent (CAA-dp) can then be deposited (**a**). Directly after CAA-dp deposition, a second layer of cells can then be printed (**b**) and incubated for 24 h. This process of depositing CAA-dp, followed by printing cells and incubating, can be cycled to establish additional layers (**c**,**d**), resulting in a 3D patterned structure (**e**). (**f**–**h**) represent a 3D patterned structure, where the base cell layer was composed of A431 cells (green), the middle layer being HaCaT cells (red), and the top layer being A431 cells (blue). A fluorescence overlay of the three colour channels is shown in (**f**). As a visualisation aid, each channel was normalised to an 8-bit greyscale image, then summed together as a 32-bit image, to readily establish where cells are layered on top of each other (**g**). A line profile was taken through (**g**), corresponding to the orange line, to generate the plot (**h**), indicating the heights after image reconstruction. The construction of a 3D liver cancer model is highlighted in (**i**–**l**). A schematic illustration of the model unit is shown in (**i**). (**j**,**k**) present fluorescence microscopy images of the second cell layer of the model unit, consisting of Hep G2 (red) and 3T3-J2 (blue) cells, images are taken at t = 0 (**j**) and 24 h after printing (**k**). As a measure of model function we compared albumin secretion among printed monoculture, 2D, and 3D tissues. Three sets of tissues (n = 3) were generated, the details of which are described in S4. The concentration levels of albumin in each set, were adjusted by subtracting the albumin concentration in the fibroblasts sample (background signal), and were normalized against albumin concentrations of monoculture tissues. Albumin production data for each model is shown as a comparison chart in (**l**). Albumin production for the 3D-printed tissues was found to be significantly higher (Student’s t-test, p-value 0.03, 95% CI) in comparison to the printed 2D tissues. Error bars represent the standard error. The scale bars in all pannels represent 300 µm.

It has been previously demonstrated that some biological tissue models show better physiological responses when cultured in a 3D environment^22^. Therefore, it is a natural advancement to apply our printing technique, to create higher-ordered, multi-layered structures. This would enable tissue assembly, via sequential cell layer deposition onto a patterned substrate, then onto each subsequent patterned cell layer. This cell printing strategy is illustrated in Fig. 3 a-d. In order to establish a multi-layered structure, a molecular-scale glue or cell attachment agent for direct patterning (CAA-dp), was developed.

Poly lysine has been used as an aid for the attachment of cells to surfaces for decades. When used in either chiral form, high molecular weight polylysine (PL) can change the surface charge. Commonly, solutions containing a few wt% of PL are deposited onto a surface, incubated at 37 °C for multiple hours, then washed and allowed to dry. This surface is then ready for use. For printing cells on to surfaces we have employed a similar strategy, using a combination of poly-l-lysine (PLL) with a dilute Geltrex (extracellular matrix solution, similar to Matrigel, details outlined in the methods section). The concentration and ratio of the PLL and Geltrex can be tuned for the specific cells which are to be adhered, however, we found that 0.5 mg/ml PLL supplemented with 75 µg/ml Geltrex (protein concentration 120–180 µg/ml), prepared using 0.1 M sodium borate buffer, to be compatible for printing most of the cell-lines used in this study.

Printing cells onto already established cell layers, requires a similar tuning of the surface properties, however, the surface to be treated in this instance, is the upper exposed layer of grown cells. 0.5 mg/ml PLL prepared in 1x phosphate buffered saline (PBS) solution containing 10% FBS, was discovered to be an appropriate CAA-dp for established cell layers, allowing cells to be printed and attach on top of other cells. CAA-dp can be loaded into the printhead and patterned onto a grown cell layer, to improve adhesion and allow multiple cell layers to be printed. CAA-dp needs to be deposited between each grown layer of cells to encourage cell–cell adhesion. CAA-dp deposition, as opposed to bulk delivery, was chosen to avoid unnecessary exposure of cells not beng printed upon, to the PLL component. This patterning strategy could be utilised to deposit cells in a layer-by-layer approach or a regional island deposition approach, in which single or a group of cells are printed into an existing natural or artificial tissue.

This use of PLL as an intercellular layer adhesive is the first demonstration, to our knowledge, of using a molecular glue to directly build up cell layers into tissues. PLL being a cationic peptide, can easily adsorb to a negatively charged surface. In our experimental setting, the PLL serves as a cationic linker between the two negatively charged cell layers. A range of 0.1–0.5 mg/ml PLL was found to be appropriate for most cell lines, with an increase of the adhesion efficiency with an increase of the PLL concentration. Concentrations in excess of 0.5 mg/ml have been observed to negatively impact the cell viability of both of the lower and upper cell layers.

A schematic and example of a multi-layered tissue is shown in Fig. 3e–h. A substrate surface is pre-cultured with a confluent cell layer of A431 cells, upon which a layer of CAA-dp was deposited, onto which a layer of HaCaT cells was printed and grown. After 24 h, a region of the HaCaT layer was patterned with CAA-dp, followed by printing A431 cells. The printing substrate chosen was a coated 500 µm gridded petridish in order to aid in identification and printing of each subsequent cell layer. Each of the cell layers was independently labelled for ease of visualisation in a multicolour fluorescence image. The base cell layer of A431 cells was labelled with cytotracker-green (green), the middle layer of HaCaT cells was labelled with cytotracker-red (red), and the top layer of A431 cells was stained with Hoechst 33342 (blue) prior to printing. A fluorescence overlay of the three colour channels (Fig. 3f) and a corresponding, normalised greyscale image (Fig. 3g) was generated to illustrate the layered nature of the printed construct. A line profile through the normalised image is presented in Fig. 3h, indicating the cell heights after image reconstruction. Images of the individual cell layers and their interpolated height comparison are presented in Supplementary Information S6 and Fig. S5.

The developed layering approach was applied to establish a liver cancer model, by printing hepatocellular carcinoma cells (Hep G2) and murine fibroblasts (3T3-J2) to form a 3D biological tissue unit (Fig. 3i–k). The generated tissues were compared to both a monoculture of the constituent cells and 2D patterns (see Supplementary Information S4, Figs. S3 and S4), by measuring the albumin production over 7 days in cell-free culture medium. The monoculture, 2D and 3D tissues contained the same bioprinted areas of Hep G2 cells, but differed in the number of fibroblasts surrounding the hepatocyte regions (Supplementary Information S4). Figure 3j–k demonstrates fluorescence microscopy images of a patch of Hep G2 cells (stained with cytotracker-red, shown in red) surrounded by 3T3-J2 cells (stained with cytotracker-green, shown in blue), which were taken at t = 0 and 24 h after printing. The base fibroblast layer was not stained for ease of visualisation. To analyse the albumin production of the tissues, four model units, such as the square presented in Fig. 3k (see also Fig. S4), were grouped together to become one sample for protein analysis (n = 3 for each experimental parameter, see Supplementary Information S4 for the experimental details). Albumin was chosen as a common liver-specific marker of hepatic cell metabolic activity^23^. We observed that the albumin secretion (based upon normalized concentration levels, see Supplementary Information S4) in 3D-printed tissues was significantly higher (Student’s t-test, p-value 0.03, 95% CI) in comparison to the printed 2D tissues, and was approximately two times higher in comparison to the Hep G2 monoculture, as shown in Fig. 3l.

## Discussion

The rapid generation of in vitro tissues for use in drug development, biological research and therapeutic approaches, requires precise control over the local cellular environment, in order to direct growth and to facilitate relevant tissue generation. The platform and process we present herein, describes an approach to address these criteria, establishing high cell deposition precision, with the ability to control the cell-to-cell ratio and the local cell pattern density.

The utility of our bioprinting approach significantly comes to light when limited material is available for building biological tissues and models; examples such as micro-biopsies, rare cell isolations, or high value patient material, all place restrictions on the sample volume. The microfluidic approach we employed, uses just 20-35 µl of cell suspension per printing run. Such a small volume is beneficial when constructing microtissues from limited cell sources, allowing much smaller tissue constructs to be generated, compared to common bioprinting approaches^2^. As an additional benefit, the cells are maintained in growth media, requiring minimal cell handling and minimising transfer losses. When working with scarce and valuable samples, such as primary neurons, access to a sufficient number can be very challenging. This can be readily appreciated when cells are required from the dorsal root ganglia, where additionally the size of the isolated neurons can vary widely, adding to the difficulty to obtain sufficient volumes of cells to print, using non-microfluidic means^24^. It is therefore of utmost importance that both the loading and dead volumes in the printing process are small, as we have achieved using the microfluidic print head.

Also, since neurons are non-dividing, it is crucial that a sufficient number of surviving cells attach to the substrate to enable downstream analysis. This is challenging to achieve due to many factors involved in the printing process such as shear stress and tuning of the printing substrate's properties since the neuronal cells’ “stickiness” to the substrate rapidly decreases after tissue extraction. Although optimisation of printing conditions is required for optimal cell attachment, the minimally invasive handling and printing procedure should be transferable to generating tissues from a much broader range of cells, such as primary human cells, and human induced pluripotent stem cells (iPSCs).

We provide tissue printing capabilities using a novel open volume microfluidics approach together with a new type of molecular-scale adhesive, where the process was developed using A431, HaCaT, Hep G2, acutely isolated rat DRG neurons, and fibroblast cells. The feasibility of building early stage tissues for use as disease models was demonstrated, where the rapid generation of contiguous cell layers is achieved from printed cells in just 24 h. Further development of this bioprinting concept in conjunction with microenvironment patterning, should lead to engineering targeted disease models in a reproducible manner. Such disease- phenotypical tissues are judged to aid in fundamental disease biology, drug discovery, and pre-clinical and clinical diagnostics. In the longer perspective, this justification can be taken even further, when connected to stem cells, as the precise positioning of the cells as well as the control of the local environment, can offer new avenues to direct differentiation, polarization, and maturation of iPSC’s, a key early requirement in establishing tissue layers for use in regenerative or therapeutic approaches.

## Materials and methods

### Solutions

Cell attachment agent (CAA) preparation for substrate pre-treatment. A working solution of 0.5 mg/ml poly-L-lysine (PLL, Sigma-Aldrich P6282) and 75 µg/ml Geltrex (Gibco, A15696-01, protein concentration 120–180 µg/ml), is prepared using 0.1 M sodium borate buffer (Alfa Aesar J60803). This solution should be prepared immediately prior to use.

Cell attachment agent for direct printing (CAA-dp) preparation. A working solution consists of 0.1 mg/ml poly-L-lysine (PLL, Sigma-Aldrich P6282) and 10% (FBS, Sigma-Aldrich F7524), prepared in a 1 × phosphate buffered saline (PBS, GE Healthcare SH30256.FS) solution. The components of CAA-dp were mixed immediately prior to use.

### Substrate surface treatment

Plastic-bottom petri dishes (ibidi (80,156) 35 mm and ibidi (81,136) 50 mm diameter) were aseptically coated with 70 µl/cm^2^ of freshly prepared CAA solution. The dishes were rocked gently to ensure even coating of the culture surface. After coating, the dishes were transferred to an incubator for 6 h at 37 °C. After 6 h, the CAA solution was removed and the surface was rinsed thoroughly with ddH_2_O water twice, finishing by removing the ddH_2_O. The dishes were dried overnight under aseptic conditions before being used for bioprinting. The substrates were batch prepared and stored in sealed antistatic bags, which were purged with nitrogen and desiccated. These sealed bags were stored at − 20 °C and have been tested (data not shown) to have no significant cell adhesion difference for up to 6 months.

For primary rat DRG cells, an alternate coating procedure was employed. Gridded 35 mm cell culture dishes (Ibidi 80156) were coated overnight at 37 °C with 0.01% poly-L-ornithine (PLO) (Sigma Aldrich P3655) in sterile water. The surface was then washed three times with ddH_2_O and coated with 3.3 µg/ml laminin (Sigma Aldrich L2020) in ddH_2_O, for 1 h at 37 °C. Laminin was removed and replaced with Neurobasal A (Gibco 10888022), supplemented with 2% B27 (Gibco 17504-044), 1% Penicillin/Streptomycin (PAA laboratories P11-010), 1% GlutaMAX (100 ×) (Gibco 35050) and 5 ng/ml rat NGF (R&D Systems 556-NG-100CF) or Neurobasal Plus (Gibco A3582901), supplemented with 2% B27 Plus (50 ×) (Gibco A3582801), 0.5 mg/ml Gentamycin (Gibco 15710), 0,25% GlutaMAX (100 ×) and 5 ng/ml rat NGF culture medium.

### Cell preparation

All cell lines were prepared using standard preparation protocols. For the details on maintaining A431 (CRL-1555, ATCC), HaCaT (T0020001, AddexBio), SK-MEL (330337, CLS), SH-SY5Y (CRL-2266, ATCC), Hep G2 (85011430, Sigma), and 3T3-J2 (P0011008, AddexBio) cells see Supporting Information S2.

When preparing cell suspensions prior to printing, Accutase (Gibco, A1110501) was used instead of Trypsin/EDTA to detach the cells from the culture flask. For printing, each cell sample was suspended to a concentration of approximately 1–10 × 10^6^ cells/ml combined with a PEG (mol wt. 6000) (Alfa Aesar A17541-30) solution, to a final PEG concentration of 15 mg/ml.

Acutely isolated primary rat DRGs were micro-surgically dissected from 5–7 weeks old male Sprague–Dawley rats in accordance with European and Swedish animal welfare regulations under ethical permit 5.8.18-11305/2018, as described elsewhere^21^. The ethics application was approved by Gothenburg Animal Research Ethics Committee. Cell number was adjusted to approximately 8 × 10^6^ cells/ml based on previous cell counts. Prior to use, the DRG suspension was adjusted to a concentration of approximately 4 × 10^6^ cells/ml, by mixing with a PEG solution.

### Cell viability tests

To analyse the viability of printed cells, multiple cell patches (each containing approximately 400 cells) were printed onto gridded petri dishes (ibidi, 80156) precoated with CAA. Cell viability was assessed at two separate timepoints; 2 h post print and 24 h post print. A live/dead staining assay, comprising of reagents containing FDA (Acros Organics, 191660050) and Propidium Iodide (Sigma-Aldrich, P4170), was employed to perform the tests. Fluorescence microscopy was used to interrogate the labelled cells, a summary of the results is presented in Table S5.

### Microscopy and fluorescence

Visualization of the cell samples was achieved using a LED fluorescence microscope (Zeiss Axiovert A1, Carl Zeiss, Germany) equipped with 5X (EC Plan-Neofluar, NA 0.16) and 10X (EC Plan-Neofluar NA 0.30) objectives, LED 385 nm, LED 470 nm and LED N-White, light sources and the corresponding filter cubes for Blue, Green and Red emission detection (Carl Zeiss filter sets Nr.49, Nr.44 and Nr.43). Images were captured using a ZEISS Axiocam 305C using Carl Zeiss, ZEN Blue v.2.6 software.

### Data analysis

Image analysis was performed using Carl Zeiss, ZEN Blue v.2.6 software and the ImageJ suite (ImageJ, U. S. National Institutes of Health, Bethesda, Maryland, USA).

### General bioprinting protocol

The bioprinting process was achieved using a similar stepwise protocol for both 2D and 3D printing. A 30 µm microfluidic printhead (width and height of the channels being 30 µm), was loaded with 25 µl of the required cell suspensions and solutions, then mounted into the holder assembly. Loading of individual printhead chambers is achieved by dispensing 25 µl of chosen cell solution using a transfer pipette. The selected substrate dish, precoated with CAA, was then filled with an amount of cell culture medium without FBS, to establish the required immersion for the printhead during printing, approximately 3 ml. The printhead was pressurised and submersed into the solution, positioning it close to the substrate surface, typically approximately 10 µm. Using the selected bioprinting protocol, which adjusts the positive and negative pressures to tune the hydrodynamically confined flow, the cells were circulated close to the substrate surface. Cells were captured by the surface as the substrate was translated to build a structure.

The choice of cell type currently being deposited can be changed, by switching the applied pressure to the chamber housing the desired cell type. Through careful pressure balancing, the choice of cell to be deposited can be readily controlled, allowing real-time choice of cell pattern density and ratio. A typical confined flow volume is 0.15 nl, with positive flow rates of 1.3 nl/s and negative flow of 12.8 nl/s. After cell printing, the substrate was transferred to an incubator (37 °C and 5% CO_2_) and allowed to attach, proliferate and/or grow until the desired cell layer was achieved. FBS was added to the growth media prior to incubation.

To build additional cell layers, the global substrate surface, including the grown cells, can be pre-treated with CAA-dp by replacing the growth medium with CAA-dp and incubating at 37 °C for 5 min. This solution is subsequently removed, and the cell laden substrate gently rinsed with culture medium without FBS. When a localised area was to be treated for printing instead of the global substrate, the CAA-dp can be locally applied by exposing the surface to the CAA-dp solution using the printhead. This localised patterning does not require a complete change of the substrate immersion medium, as with the global coating.

## Protocols relating to the Skin model (Fig. 2e,f)

### Cell culture

HaCaT cells were maintained and grown in complete Dulbecco’s Modified eagle’s Medium (DMEM, Gibco 11965-092) supplemented with 10% foetal bovine serum (FBS, Sigma-Aldrich F7524), at 37 °C and 5% CO_2_. Cells used as experimental controls were sub-cultured when they attained a confluency of approximately 80%, preventing the cells from differentiating. A separate culture of HaCaT cells was prepared and allowed to differentiate, by growing the culture for 3–4 additional days, once a 100% confluency had been achieved.

### Cytokine treatment

To induce the psoriasis-like protein expression, differentiated HaCaT cells were exposed to a combination of proinflammatory cytokines IL-1α (10 ng/ml, Peprotech 200-01A) and TNF-α (5 ng/ml, Peprotech 300-01A) for 36 h prior the bioprinting process.

### Bioprinting

Non-differentiated and differentiated (grown beyond confluency and cytokine treated) HaCaT cells were used for bioprinting a skin model. The skin model was formed by printing a two-stripe structure of non-differentiated and differentiated HaCaT cells side-by-side as a representation of a human skin section, basal and spinous layers, respectively. The printing process was performed on CAA precoated dishes (ibidi, 80156). After printing, the cells were grown and allowed to proliferate in full growth media (DMEM + 10% FBS) at 37 °C, 5% CO_2_ for 24 h.

### Retinoic acid treatment

To verify the applicability of our bioprinted skin model, we compared the effect of all-trans retinoic acid (RA) (Sigma R2625), a drug used in many skin disorders, against a control. 4 h post printing, RA was introduced to the full growth media to a final concentration of 1 µM. The model was then allowed to grow for 20 h. The control model was treated with equal amounts of the RA solvent (dimethyl sulfoxide, DMSO).

### Immunofluorescence staining

After growth, culture media was removed from the dish and the printed models were washed three times with pre-warmed (37 °C) PBS. The cells were then fixed with chilled acetone for 5–6 min at − 20 °C . Acetone was removed from the dish then thoroughly washed with PBS without allowing the cells to dry between rinses. Thereafter, fixed cells were incubated with UltraCruz Blocking reagent (*Santa Cruz Biotechnology, USA sc-516214*) for 30 min at room temperature (RT). After blocking, cells were stained with rabbit anti-human CK10 primary antibody (*Abcam ab111447*) diluted in UltraCruz Blocking reagent at RT for 1 h. Next, cells were washed three times with PBS and stained with goat anti-rabbit secondary antibody conjugated with Alexa-Fluor 488 (*Abcam ab150077*) at RT for 1 h. Afterwards, cells were washed again three times with PBS, and mounted using UltraCruz Aqueous Mounting Medium with DAPI (*Santa Cruz Biotechnology, USA sc-24941*), and sealed with coverslip.

## Printing and staining protocols relating to the primary DRG cells (Fig. 2h,i)

### Printing

25 µl of the DRG cell suspensions was loaded into a 50 µm printhead (channels size is 50 × 30 µm, width × height). For control over cell deposition a “Medium pressure” preset was chosen, and pressure settings adjusted for continuous, spatially distinct cell release. Four squares of 1 × 1 mm (2 × 2 squares) were printed and subsequently cultured for up to 9 days at 37 °C, 5% CO_2_. As controls, approximately 1.2 × 10^6^ cells were seeded in a 35 mm dish and carried along during all procedures.

### Fixation and immunostaining

The culture medium was removed, the cells were washed with 1x PBS, then fixed using a 4% PFA solution for 20 min at RT. After fixation, the cells were washed three times for 10 min with PBS solution and stored in PBS until staining. Cultures were blocked and permeabilized with 0.2% Triton-X-100 (Sigma T8787) and 2% goat serum (Invitrogen 31873) in PBS for 1 h at room temperature, incubated with primary antibodies overnight at 4 °C , washed three times for 10 min with PBS and further incubated for 2 h with secondary antibodies and Hoechst 33342 (1:10,000, Invitrogen H3570) at room temperature. A list of the used primary and secondary antibodies is included in Table S2. After washing three times for 10 min with PBS, cells were stored in PBS and the whole dish was imaged on an Operetta high content imaging system (Perkin Elmer) with 10X magnification at tubulin (ex. 649 nm/em. 666 nm), Hoechst (ex. 350 nm/em. 461 nm) and NeuN (ex. 498 nm/em. 520 nm).

## Supplementary information

Supplementary information 1

Supplementary information 2
