# Supplementary material for: 3D micro-organisation printing of mammalian cells to generate biological tissues
Source: Sci Rep. 2020 Nov 10;10:19529. doi: 10.1038/s41598-020-74191-w (PMC7656262; doi:10.1038/s41598-020-74191-w)
Supplement: Supplementary file 1 — Supplementary information 1 [file 41598_2020_74191_MOESM1_ESM.docx]

**SUPPLEMENTARY INFORMATION**

**3D micro-organisation printing of mammalian cells to generate biological tissues**

Gavin D. M. Jeffries^¤*^, Shijun Xu^¤^, Tatsiana Lobovkina^¤^, Vladimir Kirejev^¤^ Florian Tusseau^¤^, Christoffer Gyllensten^¤^, Avadhesh Kumar Singh^¤^, Paul Karila*^^^*, Lydia Moll*^^^*, Owe Orwar^§*^

^¤^*Fluicell AB, Flöjelbergsgatan 8C, 431 37 Mölndal. Sweden*

^§^ *Department of Physiology and Pharmacology, Karolinska Intitutet, Solnavägen 1, 171 77 Solna. Sweden*

*^^^Cellectricon AB, Neongatan 4B, 431 53, Mölndal. Sweden*

Correspondence to: [owe.orwar@ki.se](mailto:owe.orwar@ki.se)

**S1. Evaluation of printing precision using 2x2 cell clusters**


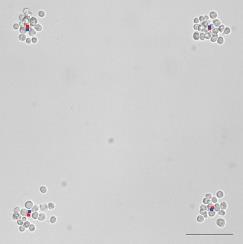


***Figure S1.*** Evaluation of printing accuracy using 2x2 cell clusters of SH-SY5Y cells (each containing approximately 15 cells). During the bioprinting process, the microscope stage was translated with the step of 500 µm in x or y direction, allowing deposition of four cell spots in the vertices of square with the side length of 500 µm, as demonstrated in Figure S1. To measure the accuracy of the cell spot deposition with respect to a square shape, an image analysis was performed. First, the input images were processed to distinguish cells from background image. After that, the flood fill algorithm was applied to identify the binary cell clusters and compute the centre of mass of each cluster (in this process, small particles, imaging artefacts and isolated cells, were excluded). The offset between the centres of mass (red spots) and the vertices of the printing square (blue spots) were used as a measure of printing accuracy. Analysis of 16 images (each containing 4 cells spots), similar to those shown in Figure S1, demonstrated that on average, the distance between the centres of the mass of the cell cluster and the vertices of the square are within 16 µm. Scale bar represents 125 µm.

**S2. Cell line culturing**

A431, HaCaT, and SK-MEL-28 cells were maintained in Dulbecco's Modified Eagle's Medium (DMEM, Gibco, 11965-092), with the addition of 1mM sodium pyruvate (Gibco, 11360-070) and 10% foetal bovine serum (FBS , Sigma-Aldrich F7524).

3T3-J2 Fibroblasts were maintained in Dulbecco's Modified Eagle's Medium (DMEM, Gibco, 11965-092) supplemented with 10% Bovine Calf Serum (BCS, Sigma-Aldrich, 12138C).

HEP G2 cells were cultured in RPMI-1640 (Gibco, A1049101) supplemented with 10% FBS (Sigma-Aldrich F7524).

SH-SY5Y cells were maintained and grown in complete 50/50 ratio Minimum Essential Medium (MEM, Gibco 41090-028)/ Ham’s F-12 Nutrient Mix (Gibco 11765-054) supplemented with 10% foetal bovine serum (FBS, Sigma-Aldrich F7524).

All cell lines were maintained at 37°C and 5% CO_2_. Cells were sub-cultured when reaching approximately 80% confluency, using Trypsin/EDTA 0.25% (w/v) 0.53 mM EDTA (Gibco, 25200-056).

**S3. Cell Viability Assays**


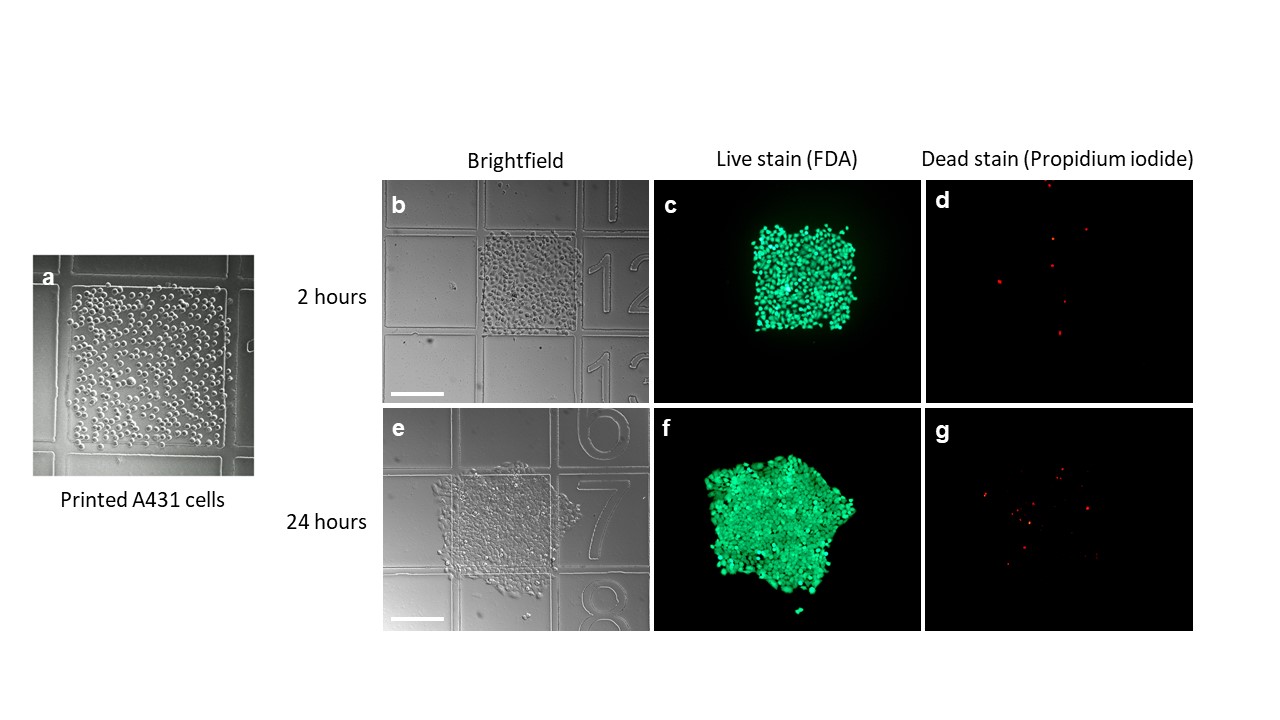


***Figure S2****.* Representative images of viability tests performed on the cell types used for bioprinting. Each assay was conducted on printed cell samples in duplicate and tested at either 2-hours or 24-hours post printing. **a**-**g**) shows a typical example of A431 cell viability tests. **a**) Brightfield image of a printed sample (0 hrs) onto a 500µm gridded coverslip. For A431, SK-MEL and HaCaT cells, one 500µm square was printed per sample (approx. 450 cells), however for Hep G2 and Fibroblasts (3T3-J2), due to their larger size, 2 squares were printed per sample (Approx. 700 cells). **b**-**d**) 2-hours post printing a sample was exposed to a live/dead staining protocol; **b**) showing a brightfield image, **c**) a green fluorescent image indicating the live cells, **d**) a red fluorescent image showing the dead cells. **e**-**g**) 24-hours post printing a sample was exposed to a live/dead staining protocol; **e**) showing a brightfield image, **f**) a green fluorescent image indicating the live cells, **g**) a red fluorescent image showing the dead cells. The scale bar in **b** & **e**) represents 300µm. Control measurements were made using the common culturing techniques for each cell type. The time points used for the Live/Dead staining were chosen to match those of the printed models. A summary of the viability data across a range of cell types is presented in Table S1.

| Cell type | Printing 2h | Traditional culture 2h | Printing 24h |
| --- | --- | --- | --- |
| A431 | 95.95% | 94.76% | 99.40% |
| HEP G2 | 97.36% | 90.03% | 86.83% |
| SK-MEL | 95.60% | 94.36% | 96.85% |
| 3T3-J2 | 99.20% | 92.84% | 98.51% |
| HaCaT | 96.70% | 97.10% | 99.54% |

***Table S1****.* Summary table comparing cell viability between printed and cultured cells.

**S4. Liver cancer model**

**
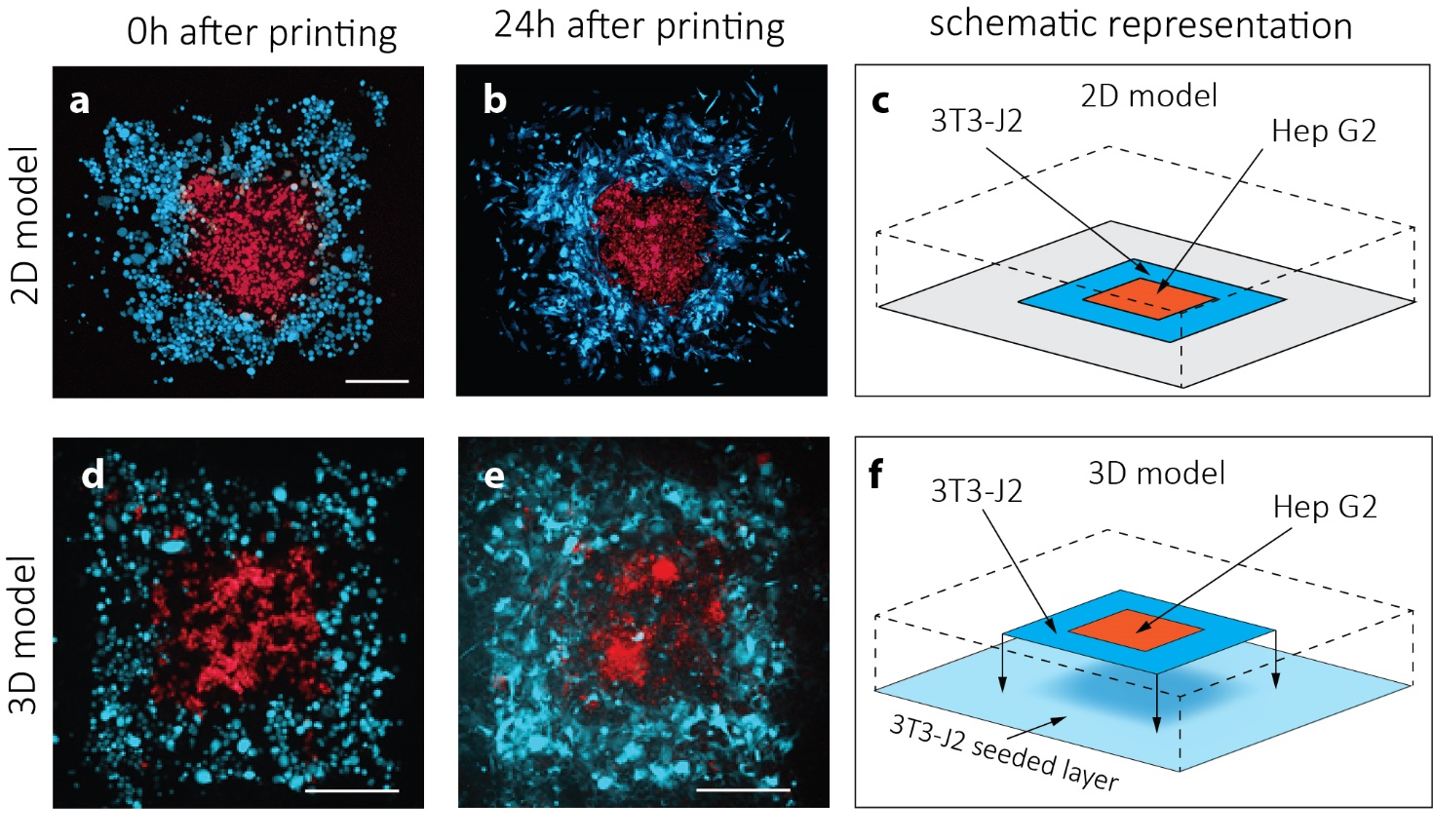
**

**Figure S3.** Liver cancer model. Fluorescence microscopy images of a printed patch of liver cancer cells (Hep G2, labelled with cytotracker-red, shown in red) surrounded by fibroblasts (3T3-J2, labelled with cytotracker-green, shown in blue) taken at t=0 and 24 hours after printing. Scale bar represents 300 µm. Upper and lower panels are 2D and 3D liver cancer models, respectively.

### **Liver cancer model: bioprinting**

To create micropatterned structures, Hep G2 cells and 3T3-J2 murine fibroblasts were printed on ibidi dishes precoated with a mixture of 0.5 mg/mL PLL (Sigma-Aldrich, P6282) and 75 µg/ml of Geltrex (Gibco, A15696-01). Cells were printed or seeded in three different combinations to generate three different types of liver cancer model: a monoculture tissue, a 2D tissue, and a 3D tissue. Each printed liver cancer model consisted of 4 units, as shown in Fig. S4. Specifically, each unit of the monoculture tissue was prepared by printing a square (500 µm x 500 µm) of Hep G2 cells. A 2D tissue unit was generated by printing a line of 3T3-J2 cells (line width approximately 250 µm) encapsulating a printed square of Hep G2 cells (500 µm x 500 µm), Fig. S3 a-c. Finally, a 3D tissue unit was prepared by printing the 2D tissue unit on top of a confluent layer of 3T3-J2 cells, thereby modulating microenvironment of hepatocytes, Fig. S3 d-f. The confluent bottom fibroblast layer was prepared by seeding in advance 3T3-J2 cells within two circular areas (of 2 mm in diameter), using PDMS inserts. To deposit the second cell layer, onto the pre-seeded fibroblast layer, first CAA-dp was coated onto the fibroblasts at the locations to be covered by the cells of the second layer. This was directly followed by printing of the second cell layer (i.e. two 2D tissue units per one circular area). Each generated tissue included the same bioprinted area of the Hep G2 cells, where the only difference between monoculture, 2D and 3D tissues was the number of 3T3-J2 cells surrounding Hep G2 regions. As a control, fibroblasts were seeded within similar circular areas (2 mm in diameter each), for measuring the albumin concentration detected in the 3T3-J2 monoculture.


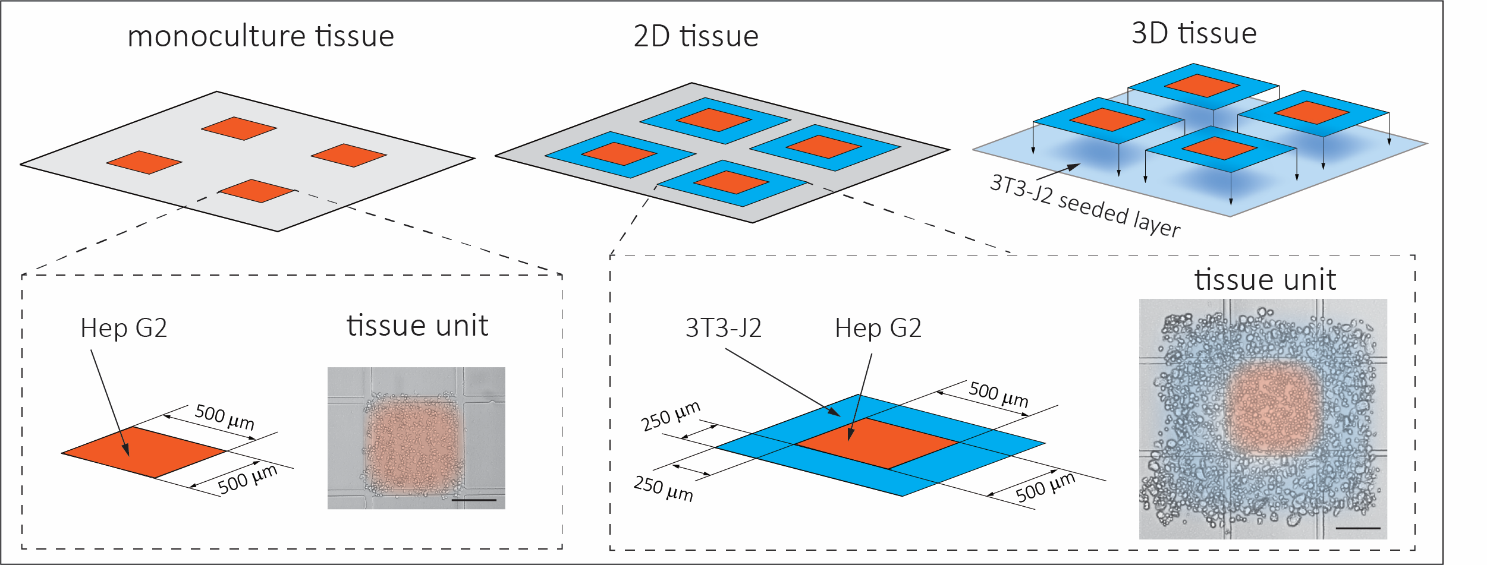
**Figure S4. Schematic illustration of bioprinted liver tissues.** The top panel presents a schematic representation of monoculture, 2D, and 3D tissues (from the left to the right respectively). The insets include schematic drawings and brightfield images of the monoculture and 2D tissue units. In the brightfield images, the regions highlighted in red and blue colours correspond to the printed areas of Hep G2 and 3T3-J2 cells, respectively. Scale bars represent 250 µm.

### **Liver cancer model: post-printing**

After printing, the models were grown and allowed to proliferate in complete Hep G2 cell growth media at 37°C, 5% CO_2_ for 7 days. Cell-free culture supernatant was collected from each dish on day 7 and immediately transferred to -20°C in multiple aliquots for future use. To quantify the secreted albumin in the tissue supernatant, ELISA was performed using albumin Human ELISA Kit (Abcam, ab108788), as per the manufacturer’s instructions.

Briefly, we used precoated 96-well plates to capture and measure albumin within each sample aliquot. Culture supernatant was thawed, diluted as described using kit buffer, and 50 µl was transferred into the wells. After 1 hr of incubation and washing, biotinylated human albumin antibody was added to the wells and incubated for 30 minutes. Following a washing step, 50 µl of streptavidin-peroxidase conjugate was added to each well and incubated for 30 minutes. The plate was washed with kit wash buffer, and 50 µl of chromogen substrate was added to each sample well. The plate was further incubated at 37°C for 25 minutes to develop the colour. After adding 50 µl of stopping solution, absorbance of the sample solution was measured using a plate reader (Multiskan™ GO Microplate, Thermo Scientific) at 450 nm. Each sample assay was performed in triplicate. All reagents and frozen culture supernatant were equilibrated to room temperature before performing the assay.

To compare albumin secretion among the printed tissues, three sets of tissues were generated, each consisting of a monoculture tissue, a 2D tissue, and a 3D tissue, Fig. S4. The printed tissues contained the same bioprinted areas of Hep G2 cells, differing only in the number of fibroblasts surrounding Hep G2 regions. Each printed set of tissues was also complemented by fibroblasts, seeded on a separate dish, covering the same surface area, as the bottom layer of fibroblasts in the 3D tissue. For each printed set *j* (*j*=1,2,3), the concentration level of albumin detected in the fibroblasts sample was denoted as $C_{F}^{(j)},$ and was used as a background level. The background-adjusted concentration of albumin of the printed tissue *i,* in set *j,* was denoted as $c_{i}^{(j)}=C_{i}^{(j)}-C_{F}^{(j)}$, where *i* =1 corresponds to the monoculture tissue, *i*=2 corresponds to the 2D-, and *i*=3 corresponds to the 3D- tissue, respectively, and $C_{i}^{(j)}$ is the measured level of albumin concentration. Within each printed set *j,*  the $c_{i}^{(j)}$ values were normalized, such that 100% of albumin production corresponded to the monoculture tissue: $x_{i}^{(j)}={(c}_{i}^{\left( j \right)}/c_{1}^{(j)})\cdot100\% i=1, 2, 3$. Figure 3l demonstrates averaged values of $x_{i}^{(j)}$, such that $\bar{x_{i}}=(\sum_{j=1}^{3} c_{i}^{\left( j \right)})/n, (i=1, 2, 3, n=3)$. The error bars are indicating standard error.

**S5. A table of the primary and secondary Antibodies used to label the primary DRG neurons.**

| Antibody | Dilution | Supplier /Lot |
| --- | --- | --- |
| Rabbit anti β-III-tubulin | 1:1000 | Sigma T2200 #028M4759V |
| Mouse anti NeuN | 1:1000 | Abcam ab104224 #GR3179189 |
| Alexa Fluor 488 goat anti-mouse | 1:1000 | Fisher Scientific A11029 #1611153 |
| Alexa Fluor 647 goat anti-rabbit | 1:1000 | Invitrogen A32733 #UC279417 |

***Table S2****.* Primary and secondary antibodies used to fluorescently label the primary DRG neurons.

**S6. Comparison between individual cell layers of the printed 3D tissue, shown in Fig. 3f.**

**
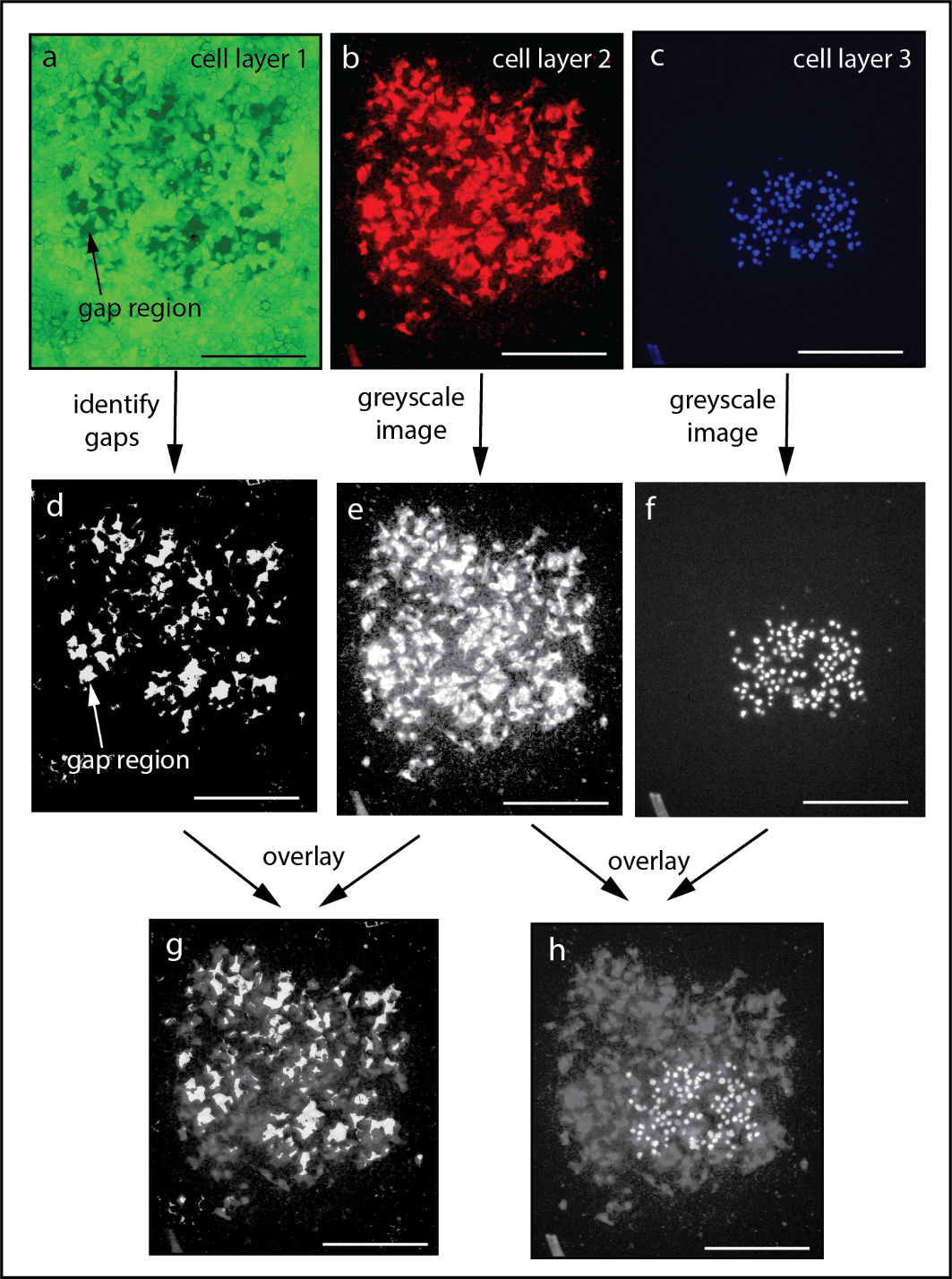
**

**Figure S5. Comparison of the individual cell layers of a 3D tissue, shown in Figure 3f. a)**, **b)** and **c)** are the fluorescence microscopy images of the first (A431, green), the second (HaCaT, red), and the third (A431, blue) cell layers, respectively. Images were taken after completing of third cell layer. **d**) is the black and white image, showing identified gaps (in white) between A431 cells within the first cell layer. **e**), **f**) are the grayscale images of the second and the third cell layers, respectively. **g**) is an overlay of the images **d** and **f**. **h**) is an overlay of the images **e** and **f**. To perform image analysis and compare different cell layers, images **a**-**c**) were transformed to an 8-bit greyscale images and analyzed using ImageJ software to generate images **d**-**h**. Scale bars represent 250 µm.

Figures S5 a-c presents fluorescence microscopy images of the individual cell layers of the printed tissue, demonstrated in Figure 3f. Within the first cell layer, shown in Fig. S5 a, we observed gaps between the A431 cells (indicated by the arrow, see also Fig. S5 d). The locations of these gaps (Fig S5 d) are overlapping with the locations of the HaCaT cells from the second layer (Fig S5 e), as it is shown in Fig. S5 g (shown by white regions). Such an overlap indicates that some of the HaCaT cells migrated from the second to the first layer. Despite of this migration, we detected a large percentage (>50% of the cell mass) of printed HaCaT cells, remained within the second layer, as indicated by the grey regions in Fig. S5 g.

Fig. S5 h demonstrates an overlay of the second (HaCat cells from Fig. S5 e) and the third (A431 cells from Fig S5 f) layers. Fig. S5 h demonstrates that the locations of A431 cells are overlapping (see white regions) with the locations of the HaCaT cells, demonstrating that A431 cells are printed on top of the HaCaT cells.

**S7. Video S1 demonstrates operation of the printhead at two different distances above the surface.**

The video S1 demonstrates operation of the printhead at two different distances above the surface using SH-SY5Y cells. The first half of the video demonstrates recirculation of the cells outside of the printhead tip when the printhead is positioned at a distance of 250 µm above the surface, resulting in no cell attachment to the surface (Positioning of the printhead with respect to the surface is presented at the start of the clip). The second half of the video demonstrates cell patterning (printing of 500 µm x 500 µm square), when the printhead tip is located at the distance of approximately 10 µm above the surface.

**S8. Printhead**


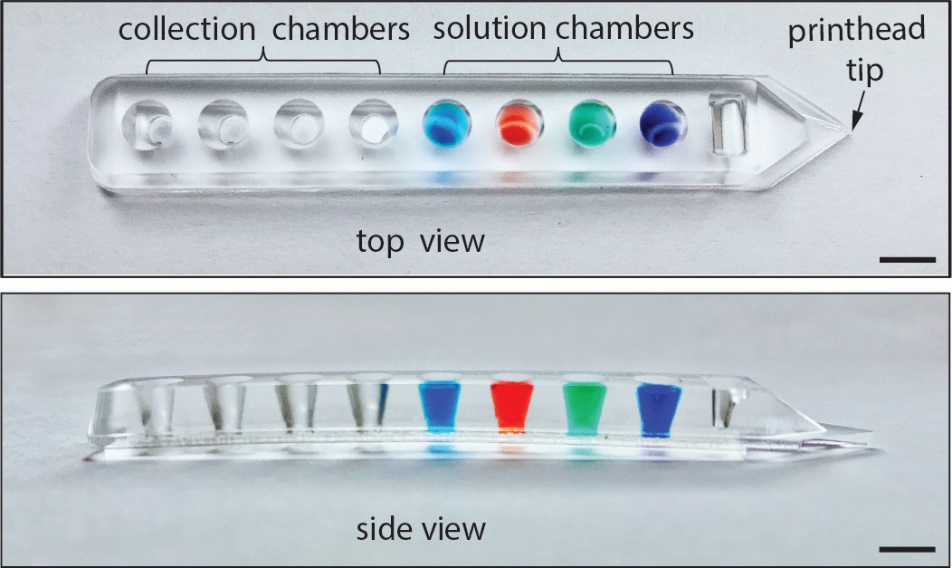


**Figure S6.** Annotated photograph of a loaded printhead. Solutions have been coloured for clarity. From right to left, the first chamber (purple) houses a washing solution or CAA-dp. The next 3 chambers (green, red and blue) house the cell suspensions. The final 4 chambers are the waste collection chambers. Scale bars represent 5mm.
